# Association of Maternal Antiangiogenic Profile at Birth With Early Postnatal Loss of Microvascular Density in Offspring of Hypertensive Pregnancies

**DOI:** 10.1161/HYPERTENSIONAHA.116.07586

**Published:** 2016-08-10

**Authors:** Grace Z. Yu, Christina Y.L. Aye, Adam J. Lewandowski, Esther F. Davis, Cheen P. Khoo, Laura Newton, Cheng T. Yang, Ayman Al Haj Zen, Lisa J. Simpson, Kathryn O’Brien, David A. Cook, Ingrid Granne, Theodosios Kyriakou, Keith M. Channon, Suzanne M. Watt, Paul Leeson

**Affiliations:** From the Division of Cardiovascular Medicine, Radcliffe Department of Medicine (G.Z.Y., C.Y.L.A., A.J.L., E.F.D., L.N., A.A.H.Z., L.J.S., K.O’B., T.K., K.M.C., P.L.), Stem Cell Research, Radcliffe Department of Medicine, Nuffield Division of Clinical Laboratory Sciences and National Health Service Blood and Transplant (G.Z.Y., C.P.K., L.N., C.T.Y., L.J.S., D.A.C., S.M.W.), Nuffield Department of Obstetrics and Gynaecology, Medical Sciences Division (C.Y.L.A., I.G.), and Wellcome Trust Centre for Human Genetics (A.A.H.Z., T.K.), University of Oxford, United Kingdom; and Peninsula Schools of Medicine and Dentistry, Plymouth University, United Kingdom (K.O’B.).

**Keywords:** embryonic ad fetal development human umbilical vein endothelial cells infant, newborn microvessels preeclampsia premature birth vascular endothelial growth factor receptor-1

## Abstract

Offspring of hypertensive pregnancies are more likely to have microvascular rarefaction and increased blood pressure in later life. We tested the hypothesis that maternal angiogenic profile during a hypertensive pregnancy is associated with fetal vasculogenic capacity and abnormal postnatal microvascular remodeling. Infants (n=255) born after either hypertensive or normotensive pregnancies were recruited for quantification of postnatal dermal microvascular structure at birth and 3 months of age. Vasculogenic cell potential was assessed in umbilical vein endothelial cells from 55 offspring based on in vitro microvessel tube formation and proliferation assays. Maternal angiogenic profile (soluble fms-like tyrosine kinase-1, soluble endoglin, vascular endothelial growth factor, and placental growth factor) was measured from postpartum plasma samples to characterize severity of pregnancy disorder. At birth, offspring born after hypertensive pregnancy had similar microvessel density to those born after a normotensive pregnancy, but during the first 3 postnatal months, they had an almost 2-fold greater reduction in total vessel density (−17.7±16.4% versus −9.9±18.7%; *P*=0.002). This postnatal loss varied according to the vasculogenic capacity of the endothelial cells of the infant at birth (*r*=0.49; *P*=0.02). The degree of reduction in both in vitro and postnatal in vivo vascular development was proportional to levels of antiangiogenic factors in the maternal circulation. In conclusion, our data indicate that offspring born to hypertensive pregnancies have reduced vasculogenic capacity at birth that predicts microvessel density loss over the first 3 postnatal months. Degree of postnatal microvessel reduction is proportional to levels of antiangiogenic factors in the maternal circulation at birth.

## Abstract 2

Supplemental Digital Content is available in the text.

A hypertensive pregnancy identifies both a mother and offspring with an increased risk of later hypertension, cardiovascular diseases, and stroke.^1–4^ The more severe the hypertensive disorder, such as occurrence of the clinical syndrome of preeclampsia,^5^ the greater the risk of later disease.^6^ Indeed, 1 in 5 of those born after more severe disorders are themselves hypertensive by the age of 20 years.^3^

Placental dysfunction is thought to be a primary event in a large proportion of women who go on to develop more severe, new-onset hypertension during pregnancy. The ischemic placenta triggers an adverse maternal circulatory milieu, including deranged angiogenic factors, inflammation, and oxidative stress.^7^ Pathophysiologically, this leads to reduced microvascular density, increased peripheral resistance, and hypertension in the mother.^8,9^ The fetus is dependent on this dysfunctional placenta,^10,11^ and, plausibly, the associated stress could have a disruptive impact on the rapidly developing fetal vascular system. Alternatively, there may be a more direct association between fetal and fetoplacental vascular development with long-term consequences for later risk of cardiovascular diseases. Consistent with this hypothesis, offspring born to preeclamptic mothers have evidence of altered endothelial cell function from very early in life^12–14^ and, in both experimental hypertensive pregnancy models and human studies, display altered microvascular structure.^15–18^ However, the timepoint when microvascular structural differences emerge and the degree to which they relate to endothelial cell dysfunction in the offspring or the severity of angiogenic markers in the mother is unclear.

Therefore, we studied whether there is a correlation between fetal vascular cell potential at birth and microvascular development, in particular, during the critical postnatal phase, as the disorderly fetal microvascular plexus remodels into the mature ex utero horizontal papillary loop structure.^19^ We then determined whether these vascular developmental differences are predicted by angiogenic markers in the maternal circulation.

## Methods

### Study Overview

**Figure 1.**
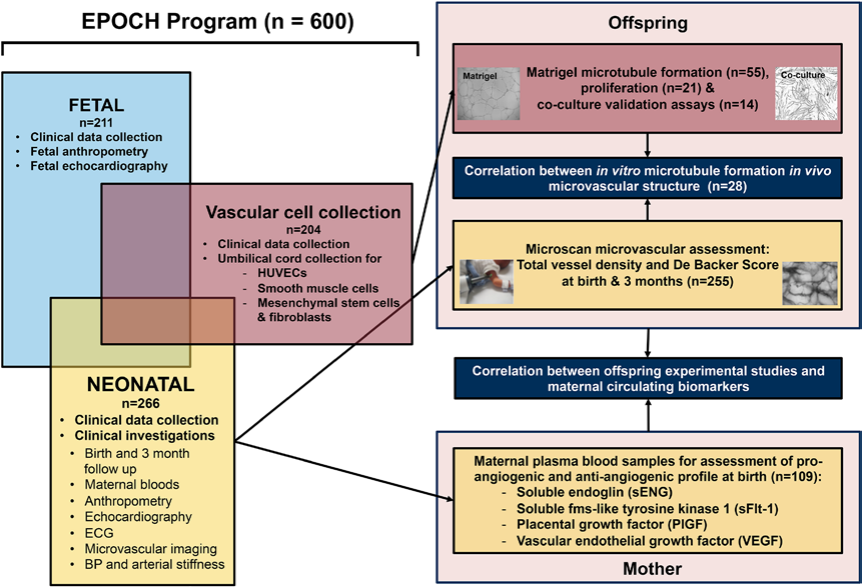
Overview of Effect of Prematurity and Hypertensive Disorders of Pregnancy on Offspring Cardiovascular Health (EPOCH) program and study design using clinical and experimental assessments in offspring born to hypertensive pregnancies. BP indicates blood pressure; HUVEC, human umbilical endothelial cell; PlGF, placental growth factor; sENG, soluble endoglin; sFlt-1, soluble fms-like tyrosine kinase-1; and VEGF, vascular endothelial growth factor.

Between 2011 and 2015, 600 mothers being cared for by Oxford University Hospitals NHS Foundation Trust were identified by their clinical care team and invited to take part in ≥1 of a portfolio of studies coordinated by the Oxford Cardiovascular Clinical Research Facility. These studies investigated associations between pregnancy complications and cardiovascular development during fetal and neonatal life (Figure 1).

To study microvascular development, we used maternal blood samples and longitudinal in vivo microvascular measures at birth and 3 months of age in the offspring recruited to the Effect of Prematurity and Hypertensive Disorders of Pregnancy on Offspring Cardiovascular Health (EPOCH) study (Ethics ref. 11/SC/0006, Clinical trials ref. NCT01888770). A stratified recruitment strategy was used in this study to recruit similar numbers of mother and infant dyads from hypertensive and normotensive pregnancies across a range of gestations and hypertensive pregnancy disorders, including pregnancy-induced hypertension and preeclampsia, defined according to International Society for the Study of Hypertension in Pregnancy guidelines^20^ (definitions are available in the Methods in the online-only Data Supplement). In addition, we performed in vitro assays using human umbilical vein endothelial cells (HUVECs) provided under ethical approval from the Oxford Cardiovascular Tissue Bioresource (ethical approval 09/H0606/68 and 07/H0606/148 ethics number 11/SC/0230). Matched in vivo measures and cell samples were available for a subset of offspring whose mothers had participated in both studies.

Mothers with a history of hypertension before pregnancy were excluded, as were infants with evidence of congenital cardiovascular disease, chromosomal abnormalities, or genetic disorders. Those with persistent features of a fetal circulation at birth, ie, a persistent ductus arteriosus or atrial septal defect, were not excluded, but the presence was recorded. All mothers gave written informed consent and assent for involvement of their children, including permission to access maternal and offspring clinical records and link data between studies. Systems for collection of clinical data and characterization of pregnancy complications based on medical records and questionnaire were standardized across studies and performed by the same data collection team (E.D., C.A., G.Y., Y.K., L.S.). Data collection details are available from https://clinicaltrials.gov (NCT0188870).

### Clinical Visit and Maternal Blood Sample Collection and Analysis

At both the birth and 3-month assessments, blood pressure and anthropometry were assessed using standard methodology (Methods in the online-only Data Supplement]. Blood samples collected from the mothers when the infants had their postnatal assessment were centrifuged and separated within 30 minutes for storage at −80°C pending analysis. Plasma circulating vascular endothelial growth factor A, soluble fms-like tyrosine kinase-1 (sFlt-1), placental growth factor, and soluble endoglin concentrations were quantified with commercial ELISAs according to standard manufacture protocols (Quantikine; R&D Systems Europe, Abingdon, United Kingdom; Methods in the online-only Data Supplement). To confirm measures at this early postnatal time point reflected levels during late pregnancy, we additionally measured the angiogenic factor sFlt-1 in a group of women who had samples collected at 34 weeks of gestation and 5 days postnatally. A subgroup of them also had measures at 3 months postnatally.

### In Vivo Microvascular Imaging

**Figure 2.**
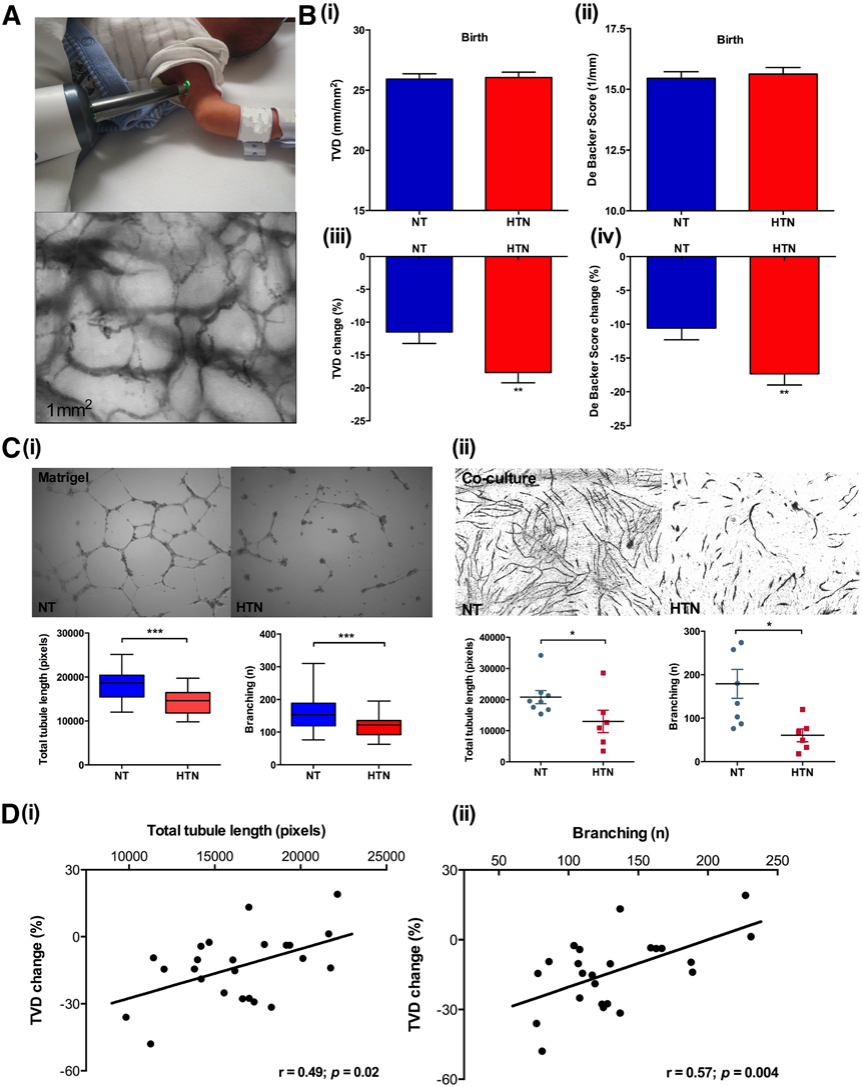
**A**, Representative images of the in vivo microscan assessment. Individual image of microscan assessment is measured of size at 1 mm × 1 mm. **B**, Total vessel density (i) and De Backer scores (ii) are similar in offspring born to normotensive and hypertensive pregnancies at birth. Reduction in total vessel density (iii) and De Backer scores (iv) between birth and 3 mo is greater in the hypertensive group. **C**, (i) Representative images of umbilical derived endothelial tubules in growth factor-reduced Matrigel. Cells were placed on Matrigel at 1×10^4^ human umbilical vein endothelial cells (HUVECs) per well in triplicates on a 96-well plate, and cultured with endothelial cell growth medium (EGM-2) for 16 h. Images were taken at ×4 magnification. Quantification of total tubule length and branching formed by normotensive and hypertensive HUVECs in Matrigel were measured using AngioSys (n=55, pixels), HUVECs were used at passage 2. (ii) Representative images of coculture assay using normotensive and hypertensive HUVECs and bone marrow stromal mesenchymal stem cells (MSCs). HUVECs and bone marrow stromal MSCs were directly cocultured at 1:5 cell ratio for 14 d in a flat bottom collagenase coated 48-well plate (n=14). Each data point represents the average (in triplets) total tubule length or branch point of one subject. HUVECs were used at passage 2. **D**, In vitro tubule network measurements (total tubule length [i] and branching [ii]) correlate with in vivo microvasculature at 3 mo using matching samples from the same infant (n=24). DB score indicates De Backer score; HTN, offspring/HUVECs from hypertensive pregnancies; NT, offspring/HUVECs from normotensive pregnancies; and TVD, total vessel density. *P<0.05, **P<0.01, ***P<0.001. Bar plots are presented as mean±SEM.

Imaging of the axillary small-vessel network was performed with Side Stream Dark Field imaging (Microscan, Microvision Medical, Amsterdam), as previously reported for neonates^21^ (Figure 2A). Measurements were performed after birth and again at 3 months, on the same side, in a temperature-controlled room, with the infant at rest, either in their mother’s arms or in a crib (Methods in the online-only Data Supplement). Analysis was performed by 1 of the 3 operators (C.A., E.D., and C.S.) blinded to the clinical background of the clip. Coefficient of variation for intra- and interobserver variability based on 10 sequences was 4.35% (intraobserver) and 6.54% (interobserver) for total vessel density (TVD) and 4.46% (intraobserver) and 6.10% (interobserver) for De Backer (DB) score.

### In Vitro Angiogenic Capacity at Birth

Umbilical cords were collected at birth, and HUVECs were isolated and stored according to standard operating procedures (Methods in the online-only Data Supplement). Angiogenic capacity was assessed by 2 complementary approaches: tube formation assays, including Matrigel and coculture with human bone marrow stromal mesenchymal stem cells, and CyQUANT NF Cell Proliferation assay (Methods in the online-only Data Supplement).

### Statistical Analysis

Statistical analysis was performed using SPSS version 22 and GraphPad Prism 6.0 (full details are provided in the Methods in the online-only Data Supplement). A sample size of 200 for the clinical study provided 80% power at a significance level of α=0.05 to detect a difference of 0.35 SD between equal-sized groups. For the in vitro tube formation cohort, a sample size of 50 provided 80% power at a significance level of α=0.05 to detect a difference of 0.75 SD between groups.

## Results

### Microvascular Measures at Birth and 3 Months

#### Cohort Characteristics

Maternal and offspring demographic and anthropometric characteristics are presented in Tables 1 and 2 (characteristics of hypertensive subgroups are available in Table S1 in the online-only Data Supplement). Hypertensive and normotensive pregnancy groups were matched for maternal age and smoking, sex ratio, birth order, gestational age and age at birth, and follow-up assessments. Maternal body mass index and blood pressure (booking, highest, and discharge) were higher in the hypertensive group (*P*<0.001), and the infants had lower birthweight *Z* score (*P*=0.002) with greater incidence of iatrogenic delivery (*P*=0.02). Microvascular measures at both birth and 3 months were available for 197 infants. Of those initially recruited, one was excluded because of a subsequent diagnosis of Turner syndrome and, of the remainder, both sets of images were not available because of nonattendance at 1 visit or equipment failure (5 visits), unanalyzable images (6 scans), or inability to obtain image (4 scans). There were no differences in the demographic or clinical characteristics of those with or without microvascular images at both timepoints (data not presented).

**Table 1.**
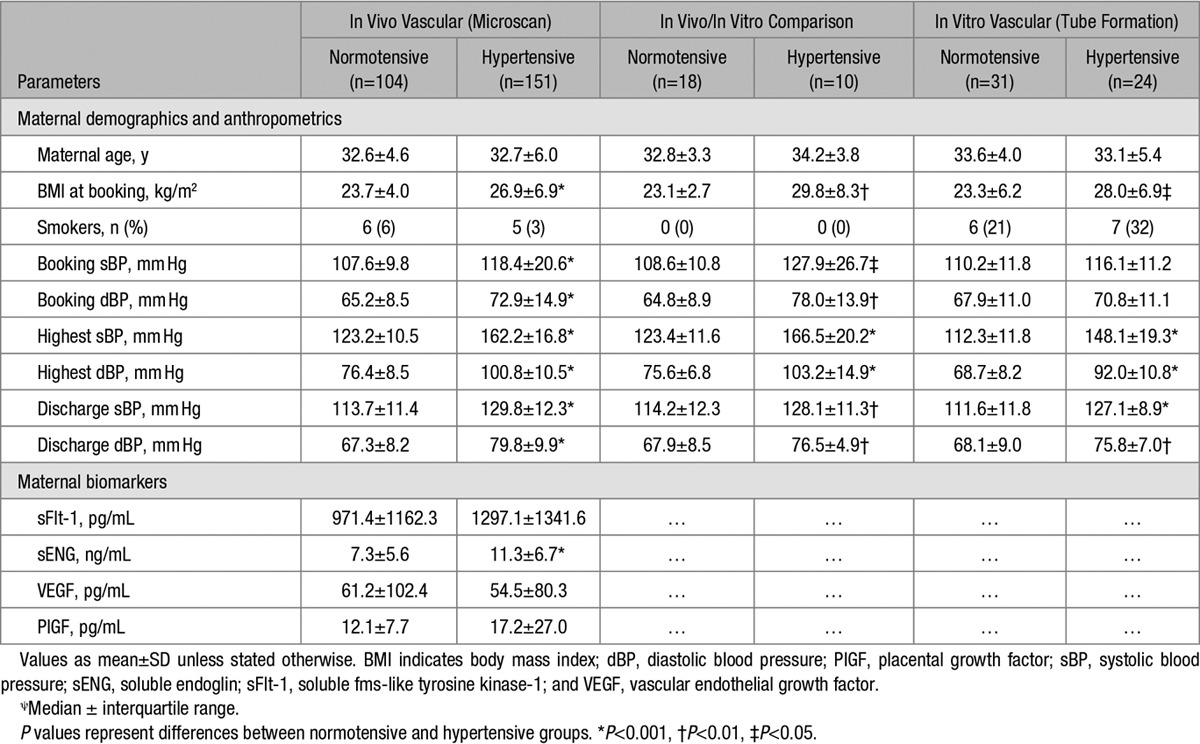
Characteristics of Cohort: Mothers

**Table 2.**
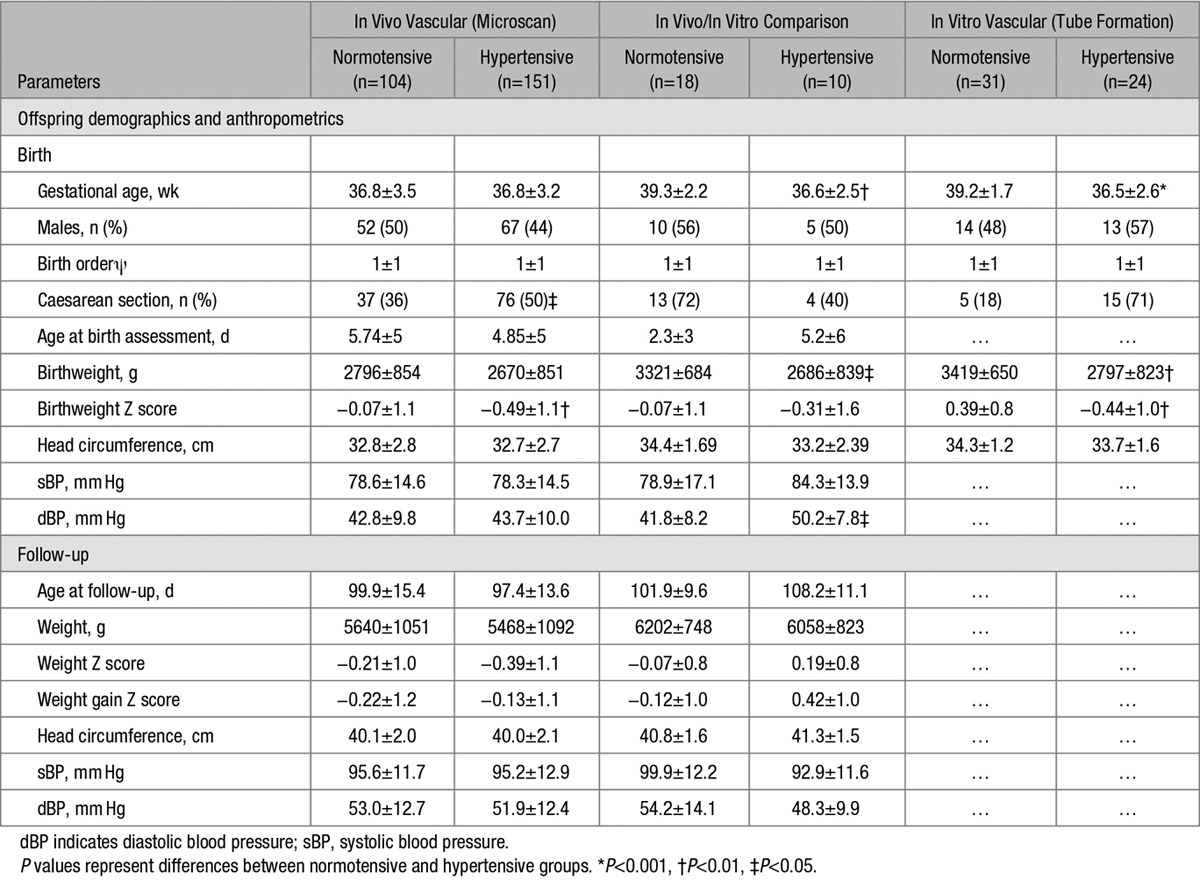
Characteristics of Cohort: Offspring

#### Microvascular Measures

There were no differences in TVD or DB score between the offspring born to normotensive and hypertensive pregnancies at birth (Figure 2B, i and ii). However, there was a significantly greater reduction in both TVD and small-vessel density as well as DB score between birth and 3 months of age in those born to hypertensive pregnancy compared with those to normotensive pregnancy (Figure 2B, results for small-vessel density not shown). The reduction in TVD and DB score between birth and 3 months was almost double in the hypertensive group compared with the normotensive group (TVD change, −9.9±18.7% versus −17.7±16.4%; *P*=0.002 and DB change, −9.6±17.2 versus −17.3±17.7; *P*=0.002) so that by 3 months, TVD was significantly lower in the hypertensive pregnancy group (21.2±4.1 versus 22.6±4.1 mm/mm^2^; *P*=0.017) with a borderline difference in the semiquantitative DB score (12.9±3.0 versus 13.6±2.6 mm^−1^; *P*=0.08).

### In Vitro Angiogenic Capacity at Birth

#### Cohort Characteristics

Maternal and fetal characteristics of the samples used for microvessel tube Matrigel measures are presented in Tables 1 and 2. As in the neonatal cohort, there was a balance of sexes between groups and maternal age, and smoking did not differ. Women with hypertensive pregnancy also tended to have slightly higher booking body mass index (*P*=0.01) and systolic and diastolic blood pressures during pregnancy. Samples available for normotensive mothers from the Cardiovascular Tissue Bioresource tended to have been collected at term, and so there were differences in gestational age (*P*=0.03) and exposure to antenatal steroids (*P*<0.001) and in higher birth weight Z score (*P*=0.002) and lower rates of iatrogenic delivery (*P*<0.001). The hypertensive pregnancy history tended to be more severe in the available tissue samples with a higher incidence of abnormal maternal liver function tests (*P*<0.001) and edema (*P*<0.001).

#### Tube Formation and Proliferation

Hypertensive pregnancy HUVECs developed a more fragmented, shorter capillary-like microvascular network in Matrigel compared with HUVECS from normotensive pregnancies (Figure 2C, i) with a significant reduction in total tubule length (hypertensive, 14438.4±2728.1; normotensive, 18239.8±3217.7 pixels; *P*<0.001) and branching (hypertensive, 118±30; normotensive, 166±49; *P*<0.001). These differences were validated in a subgroup (n=14) by coculture tube formation (Figure 2C, ii), which confirmed a reduced ability to form tube assessed by tubule length (hypertensive, 12967.2±3600.3; normotensive, 20800.1±2085.2 pixels; *P*=0.03) and branching (hypertensive, 61±15; normotensive, 179±33; *P*=0.007) compared with cells from normotensive pregnancies. Furthermore, proliferation evaluated by CyQUANT assay in a subset (n=34; figure not shown) showed hypertensive pregnancy HUVECs had significantly slower growth, presented as fold change between day 3 versus day 0, than those from normotensive pregnancies (6.3±2.0 versus 8.3±2.7; *P*=0.03).

### Correlation of In Vitro Tube Formation and In Vivo Microvasculature

Umbilical-derived cell samples and in vivo microvascular measures were available in the same individuals for 28 participants (10 hypertensive and 18 normotenisve pregnancies). In vitro tubule length and branching were not associated with in vivo measures at birth, but there was a graded positive association between in vitro total tubule length and the loss of in vivo microvascular density over the first 3 postnatal months (*r*=0.56; *P*=0.004; Figure 2D, i). Similarly, there was a graded positive association between in vitro branching and change in in vivo DB score (*r*=0.55; *P*=0.006; Figure 2D, ii).

### Severity of Hypertensive Pregnancy Disorder and Offspring Microvascular Development

#### Clinical Severity of Hypertensive Pregnancy Disorder

**Figure 3.**
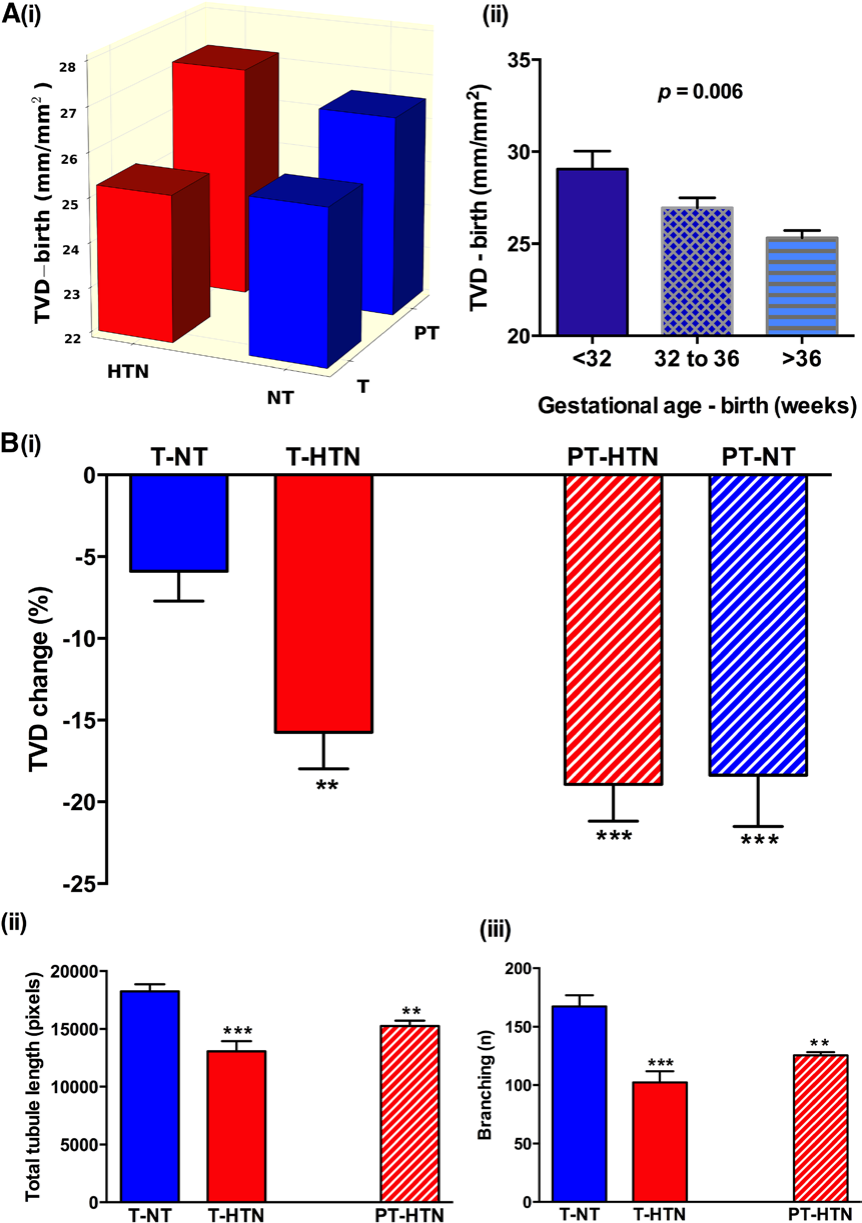
**A**, Total vessel density (TVD) is higher in offspring born preterm to both hypertensive and normotensive pregnancies compared with term pregnancies (i), and there is a graded relationship between gestational age and microvessel density at birth (ii). *P* value is presented as 1-way ANOVA test between groups. **B**, Both hypertensive and normotensive preterm offspring show a significant reduction in microvessel density during the first 3 months of life similar to that seen in term hypertensive pregnancies (i). Altered in vitro tube formation is also evident in samples from both term and preterm hypertensive pregnancy groups (ii) and (iii). PT-HTN preterm indicates hypertensive pregnancies; PT-NT, preterm-normotensive pregnancies; T-HTN term, hypertensive pregnancies; and T-NT term, normotensive pregnancies. The asterisks indicate the level of significance (*P<0.05, **P<0.01, ***P<0.001) for the 2-tailed independent sample *t* test between each pregnancy complication group with normotensive group. Bar plots are presented as mean±SEM.

In view of the association between preterm birth and hypertensive pregnancy disorders, we additionally performed analysis separately in those born term and preterm. TVD was higher in offspring born preterm to both hypertensive and normotensive pregnancies than offspring born term pregnancies (Figure 3A, i), and there was a graded relationship between gestational age and microvessel density at birth (*r*=−0.19; *P*=0.005; Figure 3A, ii). However, significant loss of microvessel density was evident in both groups compared with those born at term to normotensive pregnancy (*P*<0.001 for preterm hypertensive and *P*=0.001 for term hypertensive pregnancies; Figure 3B, i). Similar patterns were observed when hypertensive pregnancy groups were divided based on diagnosis of preeclampsia or pregnancy-induced hypertension or on the basis of diagnosis of gestation (data not shown). For in vitro measures, similar reductions in total tubule length (*P*=0.004 and *P*<0.001) and branching (*P*=0.007 and *P*<0.001) were evident in both preterm and term hypertensive pregnancies compared with term normotensive pregnancies (Figure 3B, ii and iii). In addition, responses in samples from both early and late onset preeclampsia had similar changes in tubule length and branch points compared with normotensive pregnancy samples (total tubule length, *P*=0.004 and *P*=0.006; branching, *P*=0.02 and *P*<0.001).

#### Maternal Angiogenic Profile

**Figure 4.**
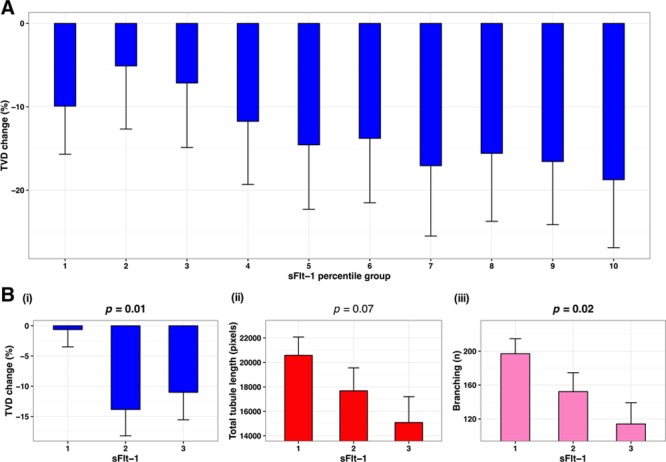
**A**, Association between maternal soluble fms-like tyrosine kinase-1 (sFlt-1) after delivery with total vessel density (TVD) % change from birth to 3 mo. Maternal sFlt-1 is presented in ten percentile groups. **B**, Comparison of maternal sFlt-1 tertiles with total vessel density (i), total tubule length (ii), and branching (iii), in normotensive pregnancies. *P* values are presented as 1-way ANOVA test between groups; *P*<0.05 are highlighted in bold; ranks are based on percentile groups with each group containing approximately the same number of cases. Bar plots are presented as mean±SEM.

Maternal postnatal blood samples were available for 107 mother and offspring dyads. Samples were collected on average 5 days after birth, at which point, maternal soluble endoglin levels were still significantly higher in hypertensive mothers with a trend for higher sFlt-1 and lower vascular endothelial growth factor (Table 1). Our study of sFlt-1 compares measures in the last trimester, to early postnatal and 3-month postnatal values. Measures at 5 days postnatally strongly correlate to late gestation values (*r*=0.85; *P*=0.007; Figure S1). By 3 months, levels had fallen to very low values and no longer reflected either late gestation or early postnatal values. Therefore, associations identified with measures at 5 days are likely to relate to variation during pregnancy. There was a graded relationship between maternal sFlt-1 level and offspring microvessel loss between birth and 3 months (Figure 4A) with higher levels predictive of greater vessel density loss (*P*=0.05). Similar relations were evident between maternal sFlt-1 level and in vitro offspring tube formation (total tubule length [*P*=0.03] and branching [*P*=0.02]; Table 3). Although similar patterns were seen with soluble endoglin levels for both vessel reduction and in vitro measures, associations were not significant. Vascular endothelial growth factor levels were not related to total vessel reduction but positively related to in vitro proliferative capacity (*P*=0.008). To study whether these associations represented coassociation with other factors linked to hypertensive pregnancy, we performed a sensitivity analysis in normotensive pregnancies. Interestingly, significant differences across tertiles of sFlt-1 in mothers who had not developed hypertension were still evident for offspring TVD change (*P*=0.01; Figure 4B, i) and in vitro tube formation (total tubule length, *P*=0.07; branching, *P*=0.02; Figure 4B, ii and iii). There were also differences in proliferation across maternal vascular endothelial growth factor levels (*P*=0.02; data not shown).

**Table 3.**
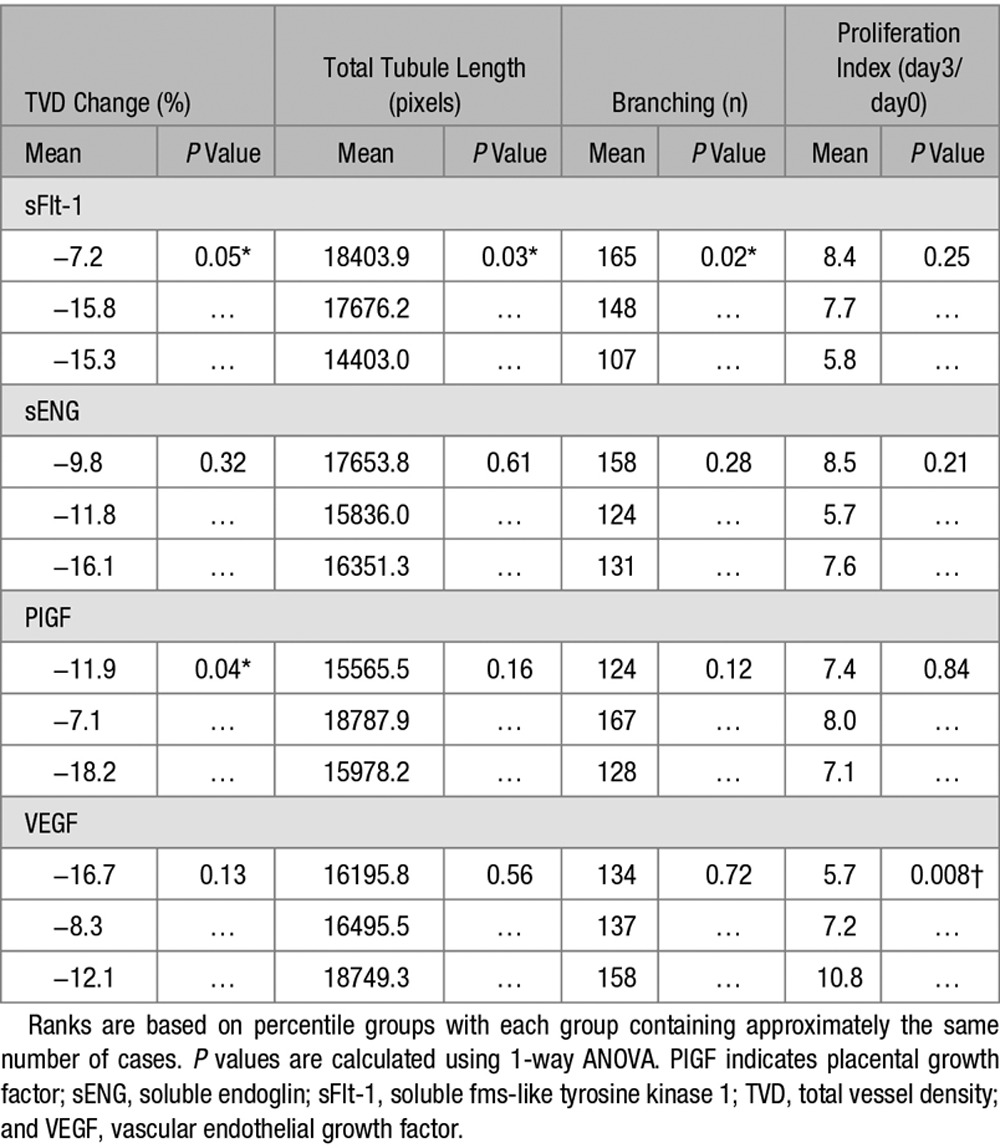
Comparison of Maternal Proangiogenic and Antiangiogenic Profile With Vascular Tubule Formation and Postnatal Microvasculature Change

### Other Predictors of Tube Formation and Microvascular Density

To study the role of other perinatal factors, we performed bivariable regression analyses with in vitro and in vivo measures (Table S2). For TVD, there were associations with blood pressure at birth, birthweight Z score, gestational age, and exposure to antenatal steroids. However, there was significant colinearity between antenatal steroid exposure and preterm birth with no differences in those born in late gestation who had received antenatal steroids compared with those who had not (data not shown). Therefore, offspring blood pressure at birth, birthweight Z score, and gestational age were taken forward into a multivariate model with maternal hypertension (Table 4). In this model, maternal hypertension remained a significant independent predictor of greater TVD change along with more preterm birth and lower blood pressure at birth. For in vitro measures, clinical markers of preeclampsia including liver function test abnormalities and edema related to tube formation, with borderline significances for gestational age, birthweight Z score, and preeclampsia-associated iatrogenic delivery. In multivariate modeling, only maternal hypertension remained an independent predictor for total tubule length (B=−0.66; *P*=0.006) and branching (B=−0.55; *P*=0.03).

**Table 4.**
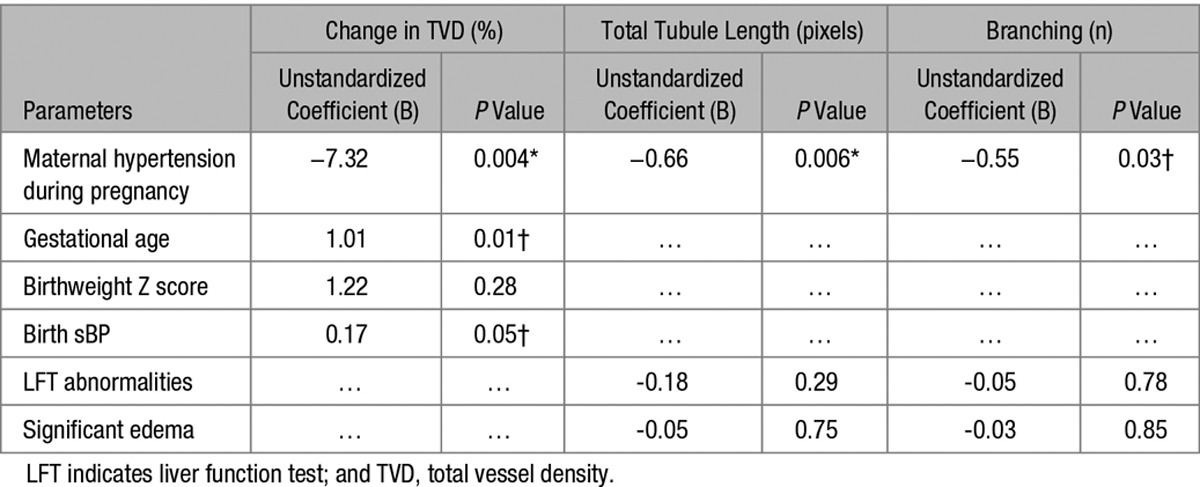
Multivariable Regression Coefficients for Maternal and Perinatal Characteristics and Microvascular Measures

## Discussion

This study provides the first evidence of an ≈2-fold greater postnatal reduction in microvascular density in neonates born after pregnancies complicated by new onset hypertension compared with normotensive pregnancies. Degree of microvessel loss was predicted by the vasculogenic capacity of their endothelial cells at birth, which, in turn, related to maternal angiogenic factors. These phenomena were evident across the spectrum of hypertensive pregnancy disorders and not attenuated by an increased vascular density in those born preterm. Intriguingly, associations were present in normotensive pregnancies if maternal antiangiogenic factors had been higher around the time of birth. These findings identify a critical postnatal microvascular developmental window and provide a potential explanation for how pregnancy pathophysiology links with microvascular rarefaction in the offspring in later life.

A small reduction in TVD during the first 3 postnatal months^19,22,23^ occurs as the disorderly fetal capillary network remodels into a horizontal subpapillary plexus and singular papillary loops structure. We hypothesized that this may be a critical period for microvascular remodeling, sensitive to inherent changes in infant endothelial cell function. Our observation that, at birth, normotensive and hypertensive pregnancy offspring had similar vessel density but then there was a loss of vessel density in the hypertensive pregnancy group and highlights the apparent importance of the postnatal window. Similar changes were seen across the spectrum of hypertensive pregnancy disorders. However, clinical diagnostic criteria may have lacked specificity for identification of offspring at the greatest risk, and maternal biomarkers may better reflect disease severity.^24^ Consistent with this hypothesis, maternal sFlt-1 after birth predicted offspring vascular postnatal changes. Strikingly, sFlt-1 associated with vascular phenotype when analysis was restricted to normotensive, full-term or appropriate for gestational age pregnancy groups. Hypertensive pregnancy syndromes may sit at one end of a spectrum,^25^ with subclinical endothelial activation, low-grade inflammation, and cardiovascular dysfunction^26^ evident in a broader group of apparently clinically normal pregnancies.^7^ Normotensive mothers who have hypertension risk factors tend to have an antiangiogenic pregnancy profile.^27–29^ Therefore, our findings may have relevance to a broader group of offspring than defined by hypertensive pregnancy alone.

On placental delivery, maternal circulating profile starts to normalize. For practical purposes, our samples were collected at the postnatal infant assessment. However, in a validation study, we were able to demonstrate measures at this timepoint, although lower, still clearly reflect levels in late gestation. The reduction in absolute levels may be why there was only a trend with soluble endoglin and timing may also explain why there was no association with placental growth factor. A recent report linked midpregnancy placental growth factor to childhood retinal vasculature,^17,28^ but differences in placental growth factor are most evident in midpregnancy.^7,24,27,28,30,31^ Biological reasons for persistent, postpartum elevation of antiangiogenic markers could be relevant to the underlying mechanisms for postnatal offspring differences as this would identify mothers with an inherent, abnormal antiangiogenic response or an extraplacental sources of sFlt-1.^32^ However, our longitudinal study of maternal sFlt-1 levels suggests that there is a general persistent elevation for several days after delivery and that values at this timepoint do not reflect baseline values measured 3 months postnatally. Nevertheless, adults born to hypertensive pregnancies have differences in circulating levels of sFlt-1 in later life,^16^ and persistent elevations in antiangiogenic factors in the infant may also be relevant. Serum from preeclamptic mothers inhibits normal HUVEC tube formation^33^ and angiogenic factors in umbilical cord blood, although significantly lower, reflect maternal levels.^10,11^ However, in our in vitro assays, with control media, tubulogenesis was still reduced, suggesting a specific alteration in cellular phenotype. As endothelial colony-forming cells account for a proportion of HUVECs,^34^ our findings may indicate a common endothelial cell dysfunctional phenotype previously described for cord blood endothelial colony-forming cells collected after preeclamptic pregnancies. These exhibited delayed colony development^13^ and, after preterm birth, were fixed in an antiangiogenic state.^35^ If so, we have linked for the first time this altered in vitro cellular behavior with in vivo vascular responses and maternal measures.

Our study is associative and does not prove causality, but our case control recruitment approach meant the number of pregnancies with complications that we could study was comparable to large population-based samples. The study size meant we could undertake detailed clinical, physiological, and sample data collection along with repeated measures over short time frames. Therefore, we could undertake detailed analysis of potential-related factors that could affect vascular development, independent of the hypertensive pregnancy. Higher skin capillary density has previously been reported in infants born preterm after hypertensive pregnancy,^18^ but our range of gestational ages demonstrated that increased vessel density is a feature of prematurity rather than hypertensive disorders. Interestingly, gestational age was also an independent predictor of postnatal microvessel loss, along with maternal hypertension, which may explain a previous observation of capillary rarefaction in adults born preterm to normotensive pregnancies.^16^ In the present study, endothelial cells were not available from normotensive preterm pregnancies to explore whether prematurity associated with in vitro cellular function, but altered cord blood endothelial colony-forming cell function has been reported in preterm offspring.^35^ In our large sample, we did not replicate the previous finding of reduced vessel density at birth in hypertensive pregnancy offspring born at term. In bivariable analysis, although maternal body mass index was not related to microvessel density change, birthweight Z score was related but, in our study, the association was not independent of a history of hypertension pregnancy and preterm birth in regression models.^36,37^ However, this factor may be of value to explore further because experimental models show that restrictive diet pregnancies can induce epigenetic cardiovascular changes.^38^ An association between low infant systolic blood pressure at birth and microvessel change persisted in multivariate analysis, which raises the possibility that complications that result in a low cardiac output or peripheral vasodilatation, for example, hypoxia or infection, may also affect postnatal microvascular development. It will be of interest to investigate whether the changes in dermal microvasculature reflect a generalized phenomenon involving other organ-specific vascular beds such as cardiac and pulmonary systems. Future longitudinal follow-up of this cohort planned over the next few years should provide definitive information on the associations between neonatal microvasculature development and emergence of cardiovascular risk evident in early life including elevated blood pressure and metabolic abnormalities.

In conclusion, we have found offspring born to hypertensive pregnancies have reduced endothelial capacity for microtubulogenesis in vitro at birth. The degree of impairment predicts the degree of reduction in vascular density during the first 3 months of postnatal life, as the fetal microvasculature remodels into its postnatal ex utero structure. Intriguingly, these changes can also be predicted by the circulating biomarker profile of the mother around the time of birth. Collectively, these findings suggest a close association between the maternal biological state during pregnancy and offspring vasculogenic capacity. As a result, although the offspring maintain microvascular structure in utero, perhaps as a compensatory response to the relative hypoxia of the hypertensive pregnancy, on transition to the normal ex utero environment, there is failure of appropriate remodeling.^39^ These changes may have long-term relevance as in adulthood offspring of complicated pregnancies are known to have microvascular rarefaction and higher blood pressure.^2,3,6,39^ Blood pressures were similar at 3 months of age in our neonates, despite reductions in vessel density, consistent with these microvascular changes being a primary event, as seen in hypertensive models and at-risk populations.^37,40^ Additional studies will help define mechanisms underlying the altered vasculogenic capacity and determine whether these changes are tractable to reduce the long-term cardiovascular and hypertensive risk.

## Perspectives

This is the first demonstration that neonates born after hypertensive pregnancies have ≈2-fold greater postnatal reduction in microvascular density compared with offspring of normotensive pregnancies and that umbilical endothelial cell tube formation predicts the degree of this postnatal microvessel reduction. Furthermore, these vascular phenotypes are predicted by levels of angiogenic factors in the maternal circulation around the time of birth, an association that is evident if the mother did not develop hypertension during pregnancy.

## Acknowledgments

We are grateful to all the pregnant women and babies who participated in this study.

## Sources of Funding

This work was supported by the British Heart Foundation (BHF; grants FS/06/024 and FS/11/65/28865 to P. Leeson; RG/13/1/30181 and CH/16/1/32013 to K.M. Channon), the NIHR Oxford Biomedical Research Centre, the Oxford BHF Centre for Research Excellence (RE/13/1/30181) and the Wellcome Trust Summer Studentship Program (109264/Z/15/Z). Support was also provided by NHS Blood and Transplant.

## Disclosures

None.

## Supplementary Material

## Novelty and Significance

### What Is New?

- Blood vessel density decreases more rapidly in offspring of hypertensive, compared with normotensive, pregnancies during early postnatal life. This seems to be predicted by how well cells from their blood vessel wall grow at birth.
- Factors that inhibit blood vessel growth are found in maternal blood during pregnancy and predict risk of hypertension during pregnancy. Higher levels in the mother also seem to predict offspring postnatal blood vessel development, even if the mother did not develop hypertension.

### What Is Relevant?

- A reduced number of blood vessels in the skin increases risk for hypertension and knowledge of maternal biological changes during pregnancy may help identify offspring who may benefit from strategies to protect vascular development during early postnatal life.

### Summary

Blood vessel wall cells in babies do not grow as effectively if their mothers had hypertension during pregnancy or had altered blood markers linked with hypertensive pregnancies, even if the maternal blood pressure was normal. These babies then have a more rapid loss of small vessels in the skin during early postnatal life, which could explain their increased risk for hypertension in later life.
