# Supplementary material for: Association of Maternal Antiangiogenic Profile at Birth With Early Postnatal Loss of Microvascular Density in Offspring of Hypertensive Pregnancies
Source: Hypertension. 2016 Aug 10;68(3):749–59. doi: 10.1161/HYPERTENSIONAHA.116.07586 (PMC4978605; doi:10.1161/HYPERTENSIONAHA.116.07586)
Supplement: Supplementary file 1 [file hyp-68-749-s001.docx]

**ASSOCIATION OF MATERNAL ANTI-ANGIOGENIC PROFILE AT BIRTH WITH EARLY POSTNATAL LOSS OF MICROVASCULAR DENSITY IN OFFSPRING OF HYPERTENSIVE PREGNANCIES**

**Online Supplemental File**

^1,2^Grace Z Yu*, PhD; ^1,3^Christina YL Aye*, BMBCh; ^1^Adam J Lewandowski, DPhil; ^1^Esther Davis, DPhil; ^2^Cheen Khoo, PhD; ^1,2^Laura Newton, BSc; ^2^Cheng Tao Yang, BSc; ^1,4^Ayman Al Haj Zen, PhD; ^1,2^Lisa Simpson, BSc (Hons); ^1,5^Kathryn O’Brien, BSc; ^2^David A Cook, PhD; ^3^Ingrid Granne PhD MRCOG; ^1,4^Theodosios Kyriakou, PhD; ^1^Keith Channon, PhD FRCP; ^2^Suzanne M Watt, PhD FRCPath; ^1^Paul Leeson, PhD FRCP *Joint first authors

^1^Division of Cardiovascular Medicine, Radcliffe Department of Medicine, University of Oxford, Oxford, UK.

^2^Stem Cell Research, Radcliffe Department of Medicine, Nuffield Division of Clinical Laboratory Sciences and NHS Blood and Transplant, University of Oxford, Oxford, UK.

^3^Nuffield Department of Obstetrics & Gynaecology, Medical Sciences Division, University of Oxford, Oxford, UK.

^4^Wellcome Trust Centre for Human Genetics, University of Oxford, Oxford, UK.

^5^Peninsula Schools of Medicine and Dentistry, Plymouth University, Plymouth, UK

Address for correspondence: Professor Paul Leeson, Oxford Cardiovascular Clinical Research Facility, Division of Cardiovascular Medicine, Radcliffe Department of Medicine, University of Oxford, John Radcliffe Hospital, Oxford. OX39DU.

E-mail: [paul.leeson@cardiov.ox.ac.uk](mailto:paul.leeson@cardiov.ox.ac.uk). Tel:+44(0)1865572846, Fax:+44(0)1865572840

Online Methods Supplement

**Definitions of pregnancy complications**

As a diastolic blood pressure > 90mmHg on two separate occasions within a 24 hour period more than three hours apart presenting after 20 weeks of pregnancy. A diagnosis of preeclampsia was also noted if there was new onset hypertension plus evidence of gestational proteinuria (300mg per 24 hours or more in a 24 hour urine collection, or at least 2+ protein at least twice on consecutive dipstick testing, or protein/creatinine ratio of ≥30mg protein/mmol creatinine). Gestational age was calculated based on first trimester ultrasound.

**Clinic Visit measures**

Weight was measured using digital scales (Charder Model MS4200) to the nearest 0.01kg with the infant fully naked. Head circumference was measured with a tape measure to nearest mm by one of two investigators trained in the techniques (CA, YK). Z-scores for weight, head circumference and weight change was calculated using preterm and term UK-WHO growth chart LMS-calculator 2010 ([www.healthforallchildren.co.uk](http://www.healthforallchildren.co.uk)). Three blood pressure measurements were recorded on right calf with an automated digital monitor (Dinamap technology® V100) using appropriate sized (2-5) SunTech® disposable neonatal cuffs with the baby lying flat either in their mother's arms or in a crib by one of three trained operators. The average of the three measurements was used.

**Maternal blood samples collection and analysis**

Plasma circulating pro-angiogenic and anti-angiogenic factors were quantified with commercial enzyme-linked immunosorbent assays (ELISAs). All samples, standards, and controls were plated in duplicate. Optical density of each well was measured at 450nm using a FLUOstar Omega microplate reader (BMG Labtech, KBioScience, USA). Data was analyzed using Omega Data Analysis software. Duplicate readings for each standard, control, and sample were averaged, and the average zero standard optical density was subtracted. Standard curves were created by generating a four-parameter logistic curve-fit. A coefficient of variation between duplicates <15% was considered acceptable.

**Microscan Imaging**

This device emits LED light at a frequency of 530 nm, which is absorbed by haemoglobin in the microvasculature to produce a dark blood image against a white/grey background for later off-line analysis. Three one-minute video clips of adjacent areas, showing a region of 1mm^2^, were recorded while the image was monitored to ensure a stable position and steady skin pressure. Analysis was performed off-line using dedicated quantitative software developed for the Microscan (AVA 3.0, MicroVision Medical). Total vessel density (TVD), small vessel density (vessels <20µm, SmVD) and De Backer (DB) score were measured for each video clip, and then the average for the three clips calculated^1^. Both are measures of capillary density. TVD is defined as the total length of capillaries per area. The DB score is a semi-quantitative measure works on the principle that the density of the vessels is proportional to the number of vessels crossing arbitrary lines^2^. In this score, three equidistant horizontal and three equidistant vertical lines are drawn on the screen. The DB score is calculated as the number of vessels crossing the lines divided by the total length of the lines. Both are derived from an inbuilt programme in the software (AVA 3.0, MicroVision Medical) after manual delineation of the capillaries in the field of view.

**Cord collection and endothelial cell isolation**

Umbilical cords were collected immediately after delivery by a dedicated research cord collection team and placed in Hanks Balanced Salt Solution (HBSS; with phenol red; PAA with 1% Penicillin/Streptomycin) to be stored at 4˚C until processing for cell isolation. All cords were processed within 12 hours of delivery. A photograph was taken to record the physical state of the cord and then any damaged or clamped areas removed. Blood clots were removed by gentle massage. One end of the vein was cannulated and secured with surgical clamping scissors to allow perfusion of 20 mL of HBSS to wash out any remaining blood. To detach the endothelial cells, the other end of the vein was cannulated and secured to allow the vein to be perfused with 20 mL of CollagenaseA (Sigma; C9722) solution (1mg/mL in HBSS with Calcium and Magnesium) and the whole cord to be incubated at 37˚C for 10 minutes. After incubation, one cannulated end of the vein was opened and the solution was collected. To improve endothelial cell yield, the vein was then perfused with an additional 30 mL HBSS, and was pooled with the previously collected 20ml of CollagenaseA solution. Cells were pelleted by centrifugation of the solution at 1,250 rpm for five minutes before seeding into a T75 culture flask in EGM-2 (Lonza) and incubated at 37˚C with 5% CO_2_. After 24 hours, fresh medium was replaced to remove any remaining non-adherent cells. EGM-2 medium was changed every two to three days until cell growth reached 70-80% confluence. Isolated cells were harvested using Accutase (PAA; L11-007) at 37˚C for five minutes and pelleted at 1,250 rpm for ten minutes. A proportion of samples were used for flow cytometry to demonstrate purity and the remainder transferred into aliquots containing 1x10^6^ cells per well and transferred for storage in liquid nitrogen.

Flow cytometry was used to characterise cell surface markers. Isolated cells were washed by fluorescence-activated cell sorting (FACS) buffer (1% BSA in DPBS, Sigma) mixing with FcR blocking buffer (BD Biosciences). Conjugated monoclonal antibodies or isotype-matched negative-controls were used for staining at a 1:10 dilution. The following monoclonal antibodies were used: PE-CD31 (mIgG1, BD; 555446), FITC-CD90 (BD; 555595), PE-Cy7-CD45 (mIgG1, BD; 345809) PE-mIgG1 isotype control, FITC-mIgG1 isotype control and PE-Cy7mIgG1 isotype control. Flow cytometry was performed using a BD LSRII flow cytometer with FACSDiva software (BD Biosciences). An unstained sample was included in each flow cytometry analysis as a negative control.

**Tube formation assays**

*Matrigel assay -* To assess tube formation ability of HUVECs, a 96-well plate was evenly coated with 50μl of growth factor-reduced Matrigel (BD Biosciences, UK) and HUVECs placed with EGM-2 (Lonza) at a density of 1x10^4^ cells per well. The plate was incubated at 37˚C for 16 hours before photomicroscopy. Each sample was replicated in triplicate and the image of each well was taken at x4 magnification using a Nikon Eclipse TE2000-U microscope (Nikon Ltd, London, UK).

*Co-culture assay for detecting tube formation* ***-*** To validate the Matrigel results we also co-cultured HUVECs with bone marrow stromal mesenchymal stem cells (MSCs) as a support for vessel maturation. Primary extracted bone marrow mesenchymal stem cells (BMMSCs) (Lonza, Cat. No. PT-2501) were cultured using mesenchymal growth media (Lonza) until reaching a sufficient number of cells for seeding a 48-well collagen coated plate, at a concentration of 2x10^4^ cells per well. The BMMSC-seeded plate was incubated at 37˚C for 24 hours before seeding HUVECs. HUVECs (either isolated from normotensive or hypertensive pregnancies) were then co-cultured on top of the BMMSCs monolayer at a ratio of 1:5. Each sample was replicated in triplicate. The cells were gently swirled to ensure an even distribution on top of the BMMSCs monolayer. Cells were cultured in EGM-2 (Lonza) media for 14 days, and the media was replaced every two days. After 14 days incubation, the media was removed and cells were washed in PBS and fixed with ice cold 70% ethanol for one hour. BSA (5%) was used to block the cells for 30 minutes. The buffer was removed before adding mouse anti-hCD31 primary antibody (1:4000) in blocking buffer (AbD Serotec; Cat No. MCA1738) before incubating on a rocker overnight at 4°C. The antibody was completely removed by washing the cells three times with PBS. Biotinylated-goat anti-Mouse IgG (1:200) was added to each well (Vector Laboratories, Cat. No. BA-9200) and incubated at room temperature for one hour. The secondary antibody was removed and washed three times with PBS followed by incubation with Vectastain Elite ABC reagent (Vector Labs Cat. No. PK-6100 series). After tertiary antibody incubation, DAB Peroxidase Substrate working solution (Vector Labs Cat. No. SK-4100) was added and incubated for ten minutes. Subsequently the cells were washed three times with dH_2_O for five minutes. The plate was air dried before images were taken using a Nikon Eclipse TS2000-U microscope.

*Image processing and tube measurements* - Images obtained from Matrigel and co-culture assays were adjusted for mean brightness using acquisition software to control the bright field illumination of the microscope (Simple PCI version 6.6.0.0; Hamamatsu corporation, Sewickley, PA). Images were saved as TIFF files, and tube formation analysed using AngioSys 1.0 (TCS Cell Works, UK). Image threshold was adjusted based on the intensity values of the monochrome image and each image then skeletonized to reduce to one pixel wide. A line was drawn over each tubule and each branch point marked with a dot. The total length of lines was quantified in pixels and total number of branch points was recorded.

**Proliferation assay**

To assess the proliferation ability of HUVECs of hypertensive and normotensive cords, CyQUANT® NF Cell Proliferation Assay (Life Technologies, USA) was performed, based on measurements of cellular DNA content via fluorescent dye binding. Cells were plated in black 96-well plates (BD Biosciences, UK) at a density of 500 cells per well, and incubated in EGM-2 medium at 37^º^C in 5% CO_2_ overnight for cellular attachment. A separate plate was prepared simultaneously as a baseline control. Fluorescence intensity values were obtained following the CyQUANT Cell Proliferation Assay kit protocol for attached cells. In brief, cells were incubated with 1x CyQUANT® NF dye binding solution for 60 minutes at 37^º^C. Fluorescence intensity was measured at excitation of ~485nm and emission of ~530nm using VICTORTM fluorescence microplate reader (Perkin Elmer, Vienna, Austria). Proliferation index was reported as fold change of averages of quadruplicate samples with baseline subtraction. Cells were used for the CyQUANT assay at passage two.

**Statistical Analysis**

Comparison between groups for continuous variables was performed using a two-tailed, independent samples *t*-test for normally distributed variables, and Mann Whiney U test for non-normally distributed data. Levels of variables across the distribution of non-normally distributed variables were also analysed with the cohort ranked into thirds or tenths of the distribution. Comparison of categorical variables was performed using a Chi-Square test. Bivariable and multivariable regression models were performed using a forced entry method. Statistically significant variables from bivariable regression analysis were then included in a multivariable regression analysis. Pearson Correlation was recorded for bivariable regression analysis. Standardized Coefficients were used in the multiple regression analysis. Results are presented as mean±standard deviation for normally distributed continuous variables, and median (minimum, maximum) for non-normal distributed variables, number of observations (yes/no) with percentage in each group for categorical variables. P-values less than 0.05 were considered statistically significant.

**REFERENCE**

1. De Backer D, Hollenberg S, Boerma C, et al. How to evaluate the microcirculation: report of a round table conference. *Crit Care.* 2007;11:R101.

**Online Data Supplement**

**Table S1: Characteristics of Neonatal Cohort**

| **Parameters** | **Preterm-born**  **NT**  **(n=49)** | **Preterm-born PET**  **(n=72)** | **Term-born**  **PIH**  **(n=43)** | **Term-born**  **PET**  **(n=36)** | **Term-born**  **NT**  **(n=55)** |
| --- | --- | --- | --- | --- | --- |
| **Maternal Demographics & Anthropometrics** | |  |  |  |  |
| Age at delivery, years | 32.4±5.3 | 33.7±6.1 | 32.6±5.3 | 31.0±6.3 | 32.7±3.9 |
| BMI at booking, kg/m^2^ | 24.3±4.3 | 26.4±5.4 | 28.6±9.6 | 25.9±3.9 | 23.2±3.5 |
| Smokers, n (%) | 4 (8.2) | 3 (4.2) | 1 (2.3) | 1 (2.8) | 2 (3.6) |
| Booking sBP, mmHg | 107.3±10.8 | 116.7±27.1 | 122.0±11.0 | 117.4±12.9 | 107.8±9.0 |
| Booking dBP, mmHg | 65.6±9.0 | 72.3±18.8 | 74.0±9.4 | 72.8±11.5 | 64.9±8.0 |
| Highest sBP, mmHg | 124.2±10.6 | 166.9±19.6 | 156.2±11.9 | 160.2±12.9 | 122.4±10.4 |
| Highest dBP, mmHg | 77.2±9.1 | 103.3±12.9 | 98.6±6.2 | 98.5±8.5 | 75.7±7.9 |
| Discharge sBP, mmHg | 113.1±12.6 | 130.4±15.3 | 128.8±9.2 | 129.8±8.9 | 114.2±10.4 |
| Discharge dBP, mmHg | 66.4±8.2 | 81.1±10.3 | 79.6±10.1 | 77.8±8.7 | 68.0±8.1 |
| **Offspring Demographics & Anthropometrics** | |  |  |  |  |
| **Birth** |  |  |  |  |  |
| Gestational age, weeks | 33.6±2.09 | 34.2±2.32 | 39.7±1.05 | 38.8±1.32 | 39.7±1.37 |
| Males, n (%) | 22 (45) | 37 (52) | 12 (28) | 18 (50) | 30 (55) |
| Birth order^ψ^ | 1±(1) | 1±(1) | 1±(1) | 1±(1) | 1±(1) |
| Caesarean section, n (%) | 22 (45) | 55 (76) | 11 (26) | 10 (28) | 15 (27) |
| Age at birth assessment, days | 6.5±6.1 | 7.2±6.0 | 3.1±3.6 | 2.7±1.3 | 5.1±7.5 |
| Birthweight, grams | 2104±552 | 2018±606 | 3443±554 | 3050±457 | 3413±544 |
| Birthweight z-score | -0.50±1.1 | -0.69±1.1 | 0.075±1.1 | -0.78±1.0 | -0.09±1.0 |
| Head circumference, cms | 30.5±2.0 | 30.9±2.6 | 34.9±1.6 | 33.5±1.4 | 34.9±1.5 |
| sBP, mmHg | 73.4±14.3 | 74.3±14.9 | 82.4±13.7 | 80.5±13.2 | 82.6±13.7 |
| dBP, mmHg | 40.5±10.2 | 41.2±9.8 | 45.2±9.9 | 46.3±9.6 | 44.6±9.3 |
| **Follow up** |  |  |  |  |  |
| Age at follow up, days | 100.4±16.4 | 98.2±14.0 | 95.3±13.9 | 98.4±12.3 | 99.5±14.7 |
| Weight, grams | 5039±947 | 4913±971 | 6064±958 | 5907±913 | 6139±860 |
| Weight z-score | -0.21±1.0 | -0.64±1.1 | -0.04±1.1 | -0.27±1.0 | -0.21±1.0 |
| Weight gain z-score | -0.13±1.3 | -0.11±1.2 | -0.19±1.1 | -0.11±1.0 | -0.30±1.1 |
| Head circumference, cms | 39.0±1.9 | 39.3±2.3 | 40.9±1.6 | 40.5±1.7 | 41.0±1.7 |
| sBP, mmHg | 94.5±11.7 | 92.4±12.7 | 95.6±12.5 | 100.4±12.3 | 96.4±11.7 |
| dBP, mmHg | 52.9±13.7 | 47.4±10.9 | 54.4±12.9 | 58.3±11.2 | 53.0±12.1 |

Values as Mean±Standard Deviation unless stated otherwise. ^ψ^Median±Interquartile range. sBP systolic blood pressure; dBP diastolic blood pressure.

**Table S2: Bivariable Regression Coefficient for Maternal and Perinatal Risk Factors and Reduction in Total Vessel Density, Total Tubule Length and Branching**

| **Parameters** | **Change in TVD (%)** | | **Total Tubule Length (pixel)** | | **Branching** | |
| --- | --- | --- | --- | --- | --- | --- |
|  | **Coefficient (B)** | ***p*-value** | **Coefficient (B)** | ***p*-value** | **Coefficient (B)** | ***p*-value** |
| **Maternal Factors** |  |  |  |  |  |  |
| Age at delivery | 0.42 | 0.07 | 0.12 | 0.38 | 0.89 | 0.53 |
| BMI at booking | 0.08 | 0.69 | -0.12 | 0.43 | -0.13 | 0.39 |
| Maternal smoking  during pregnancy | -10.34 | 0.10 | -0.5 | 0.74 | -0.1 | 0.50 |
| Booking sBP | -0.04 | 0.59 | 0.02 | 0.92 | 0.04 | 0.82 |
| Booking dBP | 0.05 | 0.63 | -0.04 | 0.78 | 0.03 | 0.83 |
| Maternal hypertension during pregnancy | -7.87 | **0.002** | -0.57 | **<0.001** | -0.53 | **<0.001** |
| LFT abnormalities | -2.02 | 0.54 | -0.39 | **0.009** | -0.32 | **0.04** |
| Significant oedema | -3.06 | 0.34 | -0.33 | **0.02** | -0.30 | **0.04** |
| **Perinatal Factors** |  |  |  |  |  |  |
| Gestational age | 1.30 | **0.001** | 0.2 | 0.16 | 0.26 | 0.06 |
| Mode of delivery | 0.74 | 0.77 | -0.25 | 0.27 | -0.36 | 0.12 |
| Age at birth assessment | 0.16 | 0.46 | - | - | - | - |
| Birthweight z-score | 2.64 | **0.02** | 0.11 | 0.46 | 0.19 | 0.18 |
| Sex | -0.11 | 0.97 | -0.07 | 0.62 | -0.03 | 0.85 |
| Apgar score (5mins) | -0.03 | 0.98 | 0.02 | 0.87 | -0.03 | 0.83 |
| Antenatal steroid  exposure | -9.89 | **0.000** | -0.18 | 0.22 | -0.24 | 0.12 |
| Birth sBP | 0.25 | **0.004** | **-** | **-** | **-** | **-** |
| Birth dBP | 0.25 | **0.05** | **-** | **-** | **-** | **-** |
| Postnatal infections | -0.59 | 0.82 | - | - | - | - |
| Days of oxygen | -0.07 | 0.50 | - | - | - | - |
| Age at follow up | 0.10 | 0.26 | - | - | - | - |
| Weight gain z-score | -0.08 | 0.94 | - | - | - | - |
| Three month sBP | -0.04 | 0.67 | - | - | - | - |
| Three month dBP | -0.002 | 0.98 | - | - | - | - |

B-unstandardized coefficient with 95% confidence interval. Bolded *p*-values are statistically significant (*p*<0.05). sBP systolic blood pressure; dBP diastolic blood pressure; LFTs liver function tests.

**Online Figure Supplement**

Figure S1 (A) Maternal sFlt-1 level is reduced from pre-delivery (34 weeks, dots in blue) to post-delivery (average of five days, dots in red); and there is a significant correlation of maternal sFlt-1 measured at the two time points. (B) Change of maternal sFlt-1 level during pre-delivery (at 34 weeks), post-delivery (average of five days), and three months post-partum (average of 98 days). sFlt-1, soluble fms-like tyrosine kinase-1. Each data point represents individual ELISA measurements Dots with connection lines on Panel A and matching colour on Panel B indicate paired maternal blood sample collected at each time point.
